# Current Status of Immune Deficiency Pathway in *Tenebrio molitor* Innate Immunity

**DOI:** 10.3389/fimmu.2022.906192

**Published:** 2022-07-04

**Authors:** Ho Am Jang, Maryam Ali Mohammadie Kojour, Bharat Bhusan Patnaik, Yeon Soo Han, Yong Hun Jo

**Affiliations:** ^1^ Department of Applied Biology, Institute of Environmentally-Friendly Agriculture (IEFA), College of Agriculture and Life Sciences, Chonnam National University, Gwangju, South Korea; ^2^ Post Graduate (PG) Department of Biosciences and Biotechnology, Fakir Mohan University, Balasore, India; ^3^ Department of Biology, College of Natural Sciences, Soonchunhyang University, Asan, South Korea

**Keywords:** *Tenebrio molitor*, Imd pathway, innate immunity, antimicrobial peptides, cross-regulation

## Abstract

Yellow mealworm (*Tenebrio molitor*) is a highly beneficial beetle that serves as an excellent source of edible protein as well as a practical study model. Therefore, studying its immune system is important. Like in other insects, the innate immune response effected through antimicrobial peptides production provides the most critical defense armory in *T. molitor*. Immune deficiency (Imd) signaling is one of the major pathways involved in the humoral innate immune response in this beetle. However, the nature of the molecules involved in the signaling cascade of the Imd pathway, from recognition to the production of final effectors, and their mechanism of action are yet to be elucidated in *T. molitor* model. In this review, we present a general overview of the current literature available on the Imd signaling pathway and its identified interaction partners in *T. molitor*.

## Introduction

Insects are the most diverse group among all living organisms. They are considered to be ahead in the “evolutionary marathon” since the Devonian period owing to their ability to survive in diverse ecological habitats (1). The signature of distinct pathogenic infections from Gram-positive/-negative bacteria, viruses, fungi, and parasites along insect life cycle exert extreme evolutionary pressure that has resulted in the development of an enhanced immune system (2, 3). Unlike the mammalian hosts, insects do not have adaptive immune system to aid them in production of antibodies and various memory cells (4). In fact, the diversity and specificity of immune priming and most specifically transgenerational immune priming (TGIP) advocated to provide clues to the immunologic memory in few insects (4). Hence, they rely on innate immune responses to protect themselves against infections and maintain homeostasis, thereby adapting to their ecological niches (5, 6).

Innate immunity is highly conserved among all living organisms. Despite the fundamental differences between insects and mammals, their battle with common pathogens for millions of years has resulted in the development of similar immunity-related molecular machinery (4). This immunity is classified into cellular immunity, including phagocytosis, encapsulation, and nodulation (7, 8), and humoral immunity, which mediates clotting (9), melanin synthesis (10), and antimicrobial peptides (AMPs) production (11, 12). In insects, AMP production, the hallmark of innate immunity (13), is mainly mediated by two intracellular signaling pathways *via* nuclear factor-kappa B (NF-κB) transcription factors: (*i*) Toll pathway, whose primary role was identified in dorso-ventral axis formation in *Drosophila* embryo (14), and in Gram-positive bacterial and fungal infection-related immune responses as identified by Hoffman et al. (15), and (*ii*) immune deficiency (Imd) pathway, which plays a role against Gram-negative bacterial infections (16).

IMD protein in insects shares similarity with receptor-interacting protein (RIP) of mammalian tumor necrosis factor receptor (TNFR) (6, 17). The insect body is able to distinguish meso-diaminopimelic acid (DAP)-type peptidoglycans (PGNs) in the Gram-negative bacterial cell wall as non-self by the peptidoglycan-recognition proteins (PGRP), PGRP-LC and PGRP-LE (18, 19). The recognition of bacterial infection by PGRPs in *Drosophila* leads to the subsequent activation of the Imd pathway by recruiting the death domain-containing intracellular protein, Fas-associated protein with death domain (FADD), and caspase-8 homolog death-related ced-3/Nedd2-like (Dredd) protein. The downstream intracellular cascade transcription factor Relish is phosphorylated and translocated into the nucleus, where it binds to the transcription response elements of AMP genes (18, 20).

Among all the insects used to study immune responses and host-pathogen interactions, yellow mealworm, *Tenebrio molitor*, has become an attractive model owing to (*i*) its convenience and cost-effective breeding, (*ii*) relatively large body size benefiting researchers to collect sufficient hemolymph samples, (*iii*) identification of molecular nature of its immune response, and (*iv*) suitability for the development of potential strategies of pest control and management (4).

The Imd pathway in *T. molitor* is relatively well-established and extensively studied in the past decade, including some studies from our research group. In this review, we have highlighted the findings related to Imd signaling, mode of action of all the receptors, death domains, positive and negative regulators, and relative effectors in *T. molitor*. We also discuss numerous open-ended questions regarding PGRP-driven bacterial recognition, intracellular domain interaction with *T. molitor* inhibitor of NF-κB (IκB) kinase (IKK) complex, putative cross-talk of this signaling pathway with other immune pathways such as Toll and c-Jun N-terminal kinase (JNK), and antimicrobial specificity of final effectors, which can only be addressed by further experiments.

## A Brief History of Imd Signaling in Insects

The discovery of adaptor protein IMD in 1995 has opened new avenues related to innate immunity in invertebrates (21). Initially, this pathway was assumed to be solely involved in sensing Gram-negative bacteria. The regulation of Imd signaling pathway can be attributed to components that are conserved across the invertebrate species. These components include the pathogen-associated molecular patterns (PAMPs) recognized by pattern recognition receptors (PRRs) such as PGRP-LC and PGRP-LE, the IMD, transforming growth factor-activated Kinase 1 (TAK1), FADD, the caspase-8 homolog, Death-related ced-3/Nedd-2-like protein (DREDD), the inhibitor of κB kinase (IKK) complex, and the NF-κB transcription factor Relish (22). In insects such as flies, mosquitoes and beetles, the PRRs such as PGRP-LC and PGRP-LE, form complexes with DAP-type PGN of Gram-negative bacteria. Alternative splicing of *PGRP-LC* results in three PGRP-LC protein isoforms (-LCa, -LCx, and -LCy) (23, 24). While PGRP-LCx is required for polymeric DAP-type PGN recognition, both PGRP-LCa and PGRP-LCx are essential to detect monomeric DAP-type PGN. PGRP-LCa, a co-receptor for PGRP-LCx, binds to the monomeric PGN fragment called tracheal cytotoxin (TCT) (25–28). PGRP-LE elicits both extra- and intracellular functions. A short form of PGRP-LE, mediates its expression on the cell surface, binds to PGN and modulates Imd signaling. In contrast, the full-length PGRP-LE is expressed in the cytoplasm, where it recognizes TCT fragments independently from PGRP-LC by directly interacting with IMD protein (29). Following recognition, the PGRPs form homo- and heterodimers, resulting in the recruitment of IMD (16). The intracellular cascade is then activated by the interaction of IMD with FADD and sequential activation of DREDD, TAK1, TAK binding protein 2 and 3 (TAB2/3), and the IKK complex (11). Subsequently, Relish is phosphorylated at multiple N-terminal sites by the IKK complex and thereafter cleaved by DREDD (30, 31). While the N-terminal transcription factor domain is released by endoproteolytic cleavage, the C-terminal part (Rel-49) remains in the cytoplasm and the active N-terminal part (Rel-68) is translocated into the nucleus, leading to the activation of antimicrobial response, elicited by the production of AMPs (32, 33). Further, IMD signaling is supplemented by TAB2, E3 ligase inhibitor of apoptosis 2 (IAP2), which associates with the E2-ubiquitin-conjugating enzymes UEV1a, Bendless (Ubc13), and Effete (Ubc5) and the transcription cofactor Akirin (22, 34).

Additional interactions of the Imd pathway with other immune signaling pathways have been reported in different insects. Evolutionary dynamics lead to various host-pathogen interactions. Therefore, insects of different orders, for instance, fruit flies, mosquitoes, and honey bees, express various immune-related genes during their interaction with pathogens. Following viral and parasitoid infections in *Drosophila*, unpaired (upd) 1, upd2, and upd3 in hemocytes bind to the dimerized Domeless receptor and activate Jak kinase (Hopscotch), resulting in phosphorylation and dimerization of STATs (Start92E) (35). Although honey bees lack upd orthologs, they can recognize viral infections *via* the same pathway and regulate relevant antimicrobial effectors such as Thioester-containing protein (TEPs) (36). Imd signaling engages with transcriptional factors after recognizing viral PAMPs. The viral patterns have been shown to stimulate REL2-regulated genes. Moreover, an specific binding sites for *D. melanogaster* NF-κB transcription factors and REL1A of *Aedes aegypti* have been found in TEP22 protein (37). Additionally, TAK1 and TAB2/3 activate the JNK pathway, leading to either the expression of AMP genes or apoptosis (36). Furthermore, phospholipase A2 (PLA2) has been identified and characterized in a wide range of animals and has diverse functions, including but not limited to host immune response. The induction of PLA2 activity in *Spodoptera exigua* is controlled by Imd signaling (38).

Imd signaling can be triggered by Gram-negative bacteria and other pathogenic sources, including fungal infections (39). Reduced survivability in Relish mutants of *D. melanogaster* and induced NF-κB REL2 in fat bodies and midgut of mosquitoes with fungal infection have been reported previously (40).

Negative regulators of Imd signaling have also been studied and identified. A membrane-bound non-catalytic PGRP-LF functions as a negative regulator of the PGRP-LC-mediated Imd pathway in *D. melanogaster* (41). Hence, some catalytic PGRPs like PGRP-SC1 and PGRP-LB are reported as negative regulator of Imd signaling *via* amidase activity against PGN (42). Moreover, in mosquitoes and honey bees, poor Imd response upon knock-in (Pirk), Rudra, and PGRP-LC-interacting inhibitor of Imd signaling (PIMS) are the other negative regulators of the pathway (35, 36). PIMS depletes the level of PGRP-LC from the plasma membrane and abrogates Imd signaling, maintaining a balanced Imd response subsequent to bacterial infections (43). The negative regulation has also been attributed to the enzyme transglutaminase that mediates cross-linking of Relish and suppresses innate immunity to commensal bacteria in the gut of *Drosophila* (44).

## Summary of Previous Reports on Imd Signaling in *T. molitor*

Despite the application of *Drosophila* as a powerful study model, using larger insects such as *T. molitor* has been benefiting researchers with more accessible biochemical investigations. As in other insects, the Imd pathway in *T. molitor* initiates an immune response by sensing invaders through PRRs such as PGRP-LC or PGRP-LE (45). Downstream of the intracellular signaling cascade, Relish enhances the production of AMPs to eliminate pathogens (46). Imd pathway components in *T. molitor* such as PGRP-LE, IMD protein, FADD, Dredd, TAK1, IKK gamma, IKK epsilon, and Relish have already been identified by our research group. Functional roles of these components have been examined using numerous pathogens, including but not limited to *Escherichia coli*, *Staphylococcus aureus*, *Candida albicans*, and *Listeria monocytogenes*, as immune elicitors. Knocking down Imd pathway components using RNA interference (RNAi) technology has shed light on various aspects, such as post-infection mortality rates and reduction in AMP levels (**Table 1**).

**Table 1:** Summary of the Imd pathway compartments that regulate antimicrobial peptide production in *T. molitor*.

Genes	Known Functions	Pathogens	Associated Organs	Regulated AMPs	References
*TmPGRP-LE*	Recognition receptor	*E. coli*	Gut	*Tm*Tene-1 *Tm*Tene-4 *Tm*Cole-A *Tm*Cole-C *Tm*Def *Tm*Cec-2 *Tm*Atta-1b *Tm*Att-2	(45)
*TmIMD*	Adapter molecule in Imd pathway	*E. coli*	Whole body	*Tm*Tene-1 *Tm*Tene-2 *Tm*Tene-4 *Tm*Def-like *Tm*Cole-A *Tm*Cole-C *Tm*Atta-1a *Tm*Atta-1b *Tm*Atta-2	(47)
*TmIKKγ*	Regulatory molecule - inhibitor of nuclear factor-κB (IκB) kinase (IKK) complex	*E. coli* *S. aureus* *C. albicans*	Fat bodies Hemocytes Gut	*Tm*Tene-1 *Tm*Tene-2 *Tm*Tene-4 *Tm*Def *Tm*Def-like *Tm*Cole-A *Tm*Cole-C *Tm*Atta-1a *Tm*Atta-1b *Tm*Atta-2	(48)
*TmIKKϵ*	Regulatory molecule - inhibitor of nuclear factor-κB (IκB) kinase (IKK) complex	*E. coli*	Fat bodies	*Tm*Tene-1 *Tm*Tene-2 *Tm*Tene-4 *Tm*Def *Tm*Def-like *Tm*Cole-A *Tm*Cole-C *Tm*Cec-2 *Tm*Atta-1a *Tm*Atta-1b *Tm*Atta-2	(49)
*TmRel*	Transcription factor (NF-κB)	*E. coli*	Fat bodies Hemocytes Gut	*Tm*Tene-1 *Tm*Tene-2 *Tm*Tene-4 *Tm*Def *Tm*Def-like *Tm*Cole-A *Tm*Cole-C *Tm*Atta-1a *Tm*Atta-1b *Tm*Atta-2	(46)

The expression of nine AMP genes (Tenecin1, Tenecin4, Attacin1a, Attacin1b, Attacin2, ColeoptericinA, ColeoptericinC, Defensin, and Defensin-like) in the insect gut reduced in response to *E. coli* infection post-*PGRP-LE* knockdown (45). Moreover, *T. molitor* larvae demonstrate an increased mortality rate post *L. monocytogenes* infection following *PGRP-LE* silencing. However, another study presented conflicting results under similar experimental conditions in a different *T. molitor* larval stage (50).

Silencing of *TmImd* increases mortality after *E. coli* and *C. albicans* infections owing to the reduced expression of nine AMP genes (Tenecin1, Tenecin2, Tenecin4, Defensin-like, ColeoptericinA, ColeoptericinC, Attacin1a, Attacin1b, and Attacin2) and five AMP genes (Tenecin2, Defensin-like, ColeoptericinA, Attacin1a, and Attacin2), respectively (47).

Likewise, IKK epsilon-silenced *T. molitor* larvae showed enhanced susceptibility post-*E. coli* infection owing to reduced expression of 12 AMP genes (Tenecin1, Tenecin2, Tenecin4, Defensin, Defensin-like, ColeoptericinA, ColeoptericinC, Attacin1a, Attacin1b, Attacin2, Thaumatin-like protein1, and Thaumatin-like protein2) in fat bodies, which are the major immune organ in insects. Reduced expression of 10 AMP genes (Tenecin1, Tenecin4, Defensin, ColeoptericinA, ColeoptericinC, Cecropin-2, Attacin1b, Attacin2, Thaumatin-like protein1, and Thaumatin-like protein2) in the gut and four AMP genes (Defensin, Defensin-like, ColeoptericinC, and Attacin2) in the hemocytes following IKK-epsilon knockdown elevated the risk of *E. coli* infection-mediated mortality (49). In addition, silencing the IKK gamma gene enhanced the susceptibility of *T. molitor* larvae to *E. coli*, *S. aureus*, and *C. albicans* infections (48). The understanding of the *T. molitor* Imd signaling cascade under pathogenic stress is still under examination. Understanding the complexity and intricate cross-talk mechanisms in response to varied pathogens would provide interesting insights of the defense mechanisms in the beetle innate immunity.

**Figure 1:**
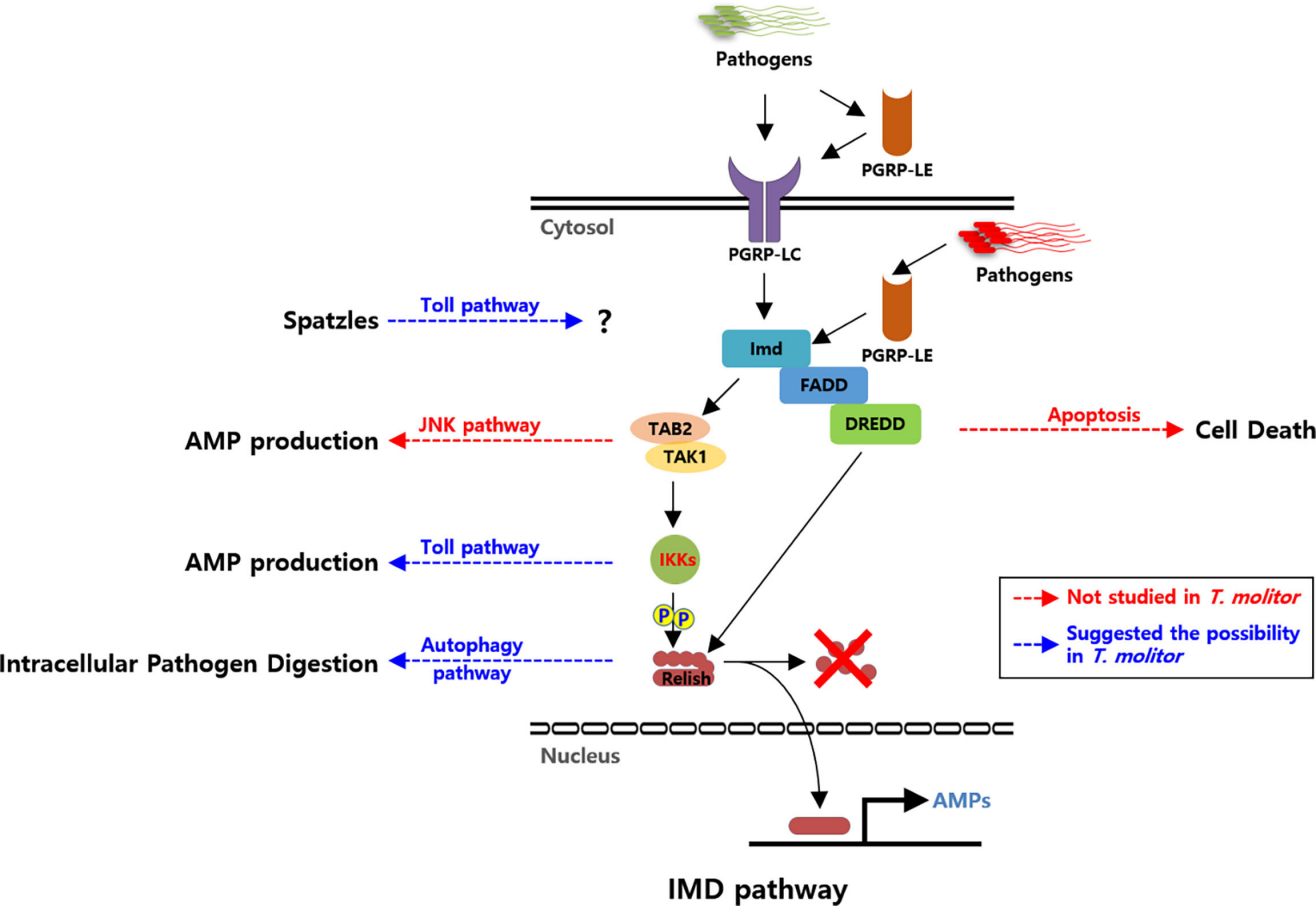
Schematic illustration of the proposed Imd pathway in *Tenebrio molitor* and its possible cross-talks. Pattern recognition receptors (PRRs; PGRP-LC and PGRP-LE) are triggered by DAP-type PGN of the bacterial cell wall. Recognition of Gram-negative bacteria further triggers the recruitment of intracellular proteins *Tm*IMD, *Tm*FADD, and *Tm*DREDD. *Tm*TAK1/*Tm*TAB2, activated by *Tm*IMD, further activates *Tm*IKK complex. *Tm*Relish is subsequently phosphorylated by the *Tm*IKK complex and then cleaved by *Tm*Dredd. Eventually, it leads to the translocation of *Tm*Relish into the nucleus, where it binds to the relevant transcription response elements and triggers AMP production. Solid black arrows indicate the identified interactions between Imd signaling compartments. Blue dashed arrows indicate putative cross-talks between Imd and other signaling pathways *via Tm*IKKs complex, *Tm*Relish, and ligand Spaetzle in *T. molitor*. Red dashed arrows indicate the putative cross-talks identified in other insects. Abbreviations: PGRP; Peptidoglycan recognition protein, IMD; Immune deficiency, FADD; Fas-associated protein with death domain, DREDD; death-related ced-3/Nedd2-like protein, TAK1; Transforming growth factor-activated kinase1, TAB2; TAK binding protein 2, IKK; Inhibitor of nuclear factor-κB (IκB) kinase, AMP; Antimicrobial peptide, JNK; c-Jun N-terminal kinase.

Further investigations on the downstream molecules in the Imd pathway and transcription factor Relish have proven the role of Imd signaling in bacterial (Gram-negative and Gram-positive) and fungal infections. For instance, in ds*TmRelish*-treated larvae, mortality of almost 90% was attributed to the downregulation of AMPs such as Tenecin3, Tenecin4, ColeoptericinA, and Attacin1a in all tissues. Hence, direct interaction of Relish and production of AMPs against *E. coli* infection in *T. molitor* support the role of Imd signaling in the host-mediated immune response (46). Additionally, Relish plays a critical role in inducing autophagy-related genes against *L. monocytogenes* infection in the fat bodies and hemocytes of *T. molitor* (51) (**Figure 1**).

## Cross-Regulation of Imd and Toll Pathways in *T. molitor*

Cross-regulation of Imd and Toll pathways have been previously documented in various insects, such as *Drosophila*, *Tribolium castaneum*, and *Plautia stali* (20). Studies on *T. molitor* also provided evidence for the cross-regulation of Imd and Toll pathways (**Figure 1**). Generally, insects elicit distinct immune responses depending on the pathogen source. For instance, Toll signaling in *Drosophila* can be activated solely after recognizing lysine-type PGN of Gram-positive bacteria or fungal beta-1,3-glucan (52). In contrast, the Imd pathway is activated simply by recognizing DAP-type PGN of Gram-negative bacteria or certain Gram-positive bacilli (53). However, in *T. molitor*, polymeric DAP-type PGN of Gram-negative bacteria can trigger both Imd and Toll pathways (54). The studies that have proposed the intracellular cross-regulation between these two signaling pathways are listed in **Table 2**.

**Table 2:** Potential evidence for the interactions between Imd pathway and other immune signaling pathways in *T. molitor*.

Genes	Signaling pathway	Pathogens	Associated Organs	Effects on the other immune pathway	References
*TmPGRP-LE*	Autophagy	*L. monocytogenes*	Whole body	decreased larval survivability	(50)
*TmIKKγ*	Toll	*E. coli* *S. aureus* *C. albicans*	Fat bodies Hemocytes Gut	Positive regulation of *Tm*DorX2	(48)
*TmIKKϵ*	Toll	*E. coli*	Fat bodies Gut	Positive regulation of *Tm*DorX2	(49)
*TmRel*	Autophagy	*L. monocytogenes*	Fat bodies	Positive regulation of *Tm*Atg1 and *Tm*Vps34	(51)
			Gut	Positive regulation of *Tm*Vps34, *Tm*Atg9, *Tm*Atg5, and *Tm*Atg8	

Knockdown of IKK gamma causes decreased survivability after *E. coli*, *S. aureus*, and *C. albicans* infections. IKK gamma silencing resulted in the downregulation of Transcription factors Relish and DorX2-encoding genes downstream of Imd and Toll pathways, respectively. Consequently, the gene expression of ten relevant AMPs was also suppressed. Concurrently, DorX1 expression was upregulated, suggesting that IKK gamma can act as a positive and negative regulator of Toll and Imd signaling pathways (48). Furthermore, IKK epsilon, another unit of the IKK complex, was involved in the expression of three NF-κBs (DorX1, DorX2, and Relish) and AMPs in fat body tissues. These results suggest that IKK epsilon plays a pivotal role in regulating Toll and Imd pathways in the fat body tissues of *T. molitor*. Nevertheless, the survivability of larvae was not affected by the invasion of *S. aureus* and *C. albicans* post-IKK epsilon knockdown, whereas they showed susceptibility after *E. coli* infection (49).

Additionally, lysine-type PGN of Gram-positive bacteria in *Drosophila* can be sensed by PGRP-SA and Gram-negative binding protein 1 (GNBP-1) (15). In contrast, various studies have clarified that PGRP-SA of *Bombus ignitus*, *Apis mellifera*, and *Megachile rotundata* tended to bind to DAP-type PGN rather than lysine-type PGN (55). In *T. molitor*, PGRP-SA plays an important role in survivability against bacterial (Gram-negative and Gram-positive) and fungal infections (56). Furthermore, Toll receptor can be activated by its ligand, Spaetzle (Spz). This protein is a zymogen and is cleaved to its mature form by a chain of serine protease activation, following pathogen recognition by PRRs (24, 57). The immunological roles of Spz isoforms (Spz1b, Spz-like, Spz4, Spz5, and Spz6) in *T. molitor* have been investigated using RNAi (58–62). Among them, *Tm*Spz1b, *Tm*Spz-like, and *Tm*Spz5 showed anti-Gram-negative bacterial (*E. coli*) activity (58, 61, 62). Moreover, silencing of *TmSpz1b* downregulated the expression of *TmDorX1* and *TmRel* in the immune organs (58). Similarly, *TmSpz-like* knockdown suppressed the expression of all NF-κB genes (61). The *TmSpz5*-silenced larvae, however, showed a decreased expression of *TmRel* after Gram-negative bacterial infection but an increased expression of the same after a Gram-positive bacterial infection in the Malpighian tubules (62). Collectively, the activation of either Toll or Imd signaling pathways interferes with the other through unknown interactions between their components (**Table 3**).

**Table 3:** Summary of Toll pathway compartments affecting the regulation of Imd signaling in *T. molitor*.

Genes	Pathogens	Activating Organs	Effects on Imd pathway	References
*TmPGRP-SA*	*E. coli*	Fat bodies Gut	Positive regulation of *Tm*Relish	(56)
*TmSpz1b*	*E. coli*	Fat bodies Hemocytes	Positive regulation of *Tm*Relish	(58)
*TmSpz-like*	*E. coli*	Whole body	Positive regulation of *Tm*Relish	(61)
*TmSpz5*	*E. coli*	Malpighian tubules	Positive regulation of *Tm*Relish	(62)
	*S. aureus*	Malpighian tubules	Negative regulation of *Tm*Relish	

Another pathway that supposedly interacts with Imd signaling is autophagy, a conserved cellular mechanism that maintains homeostasis by eliminating dysfunctional cellular components and intracellular pathogens mediating its delivery to the lysosomes (63, 64). PGRP-LE recognizes the intracellular pathogen *Listeria* and induces autophagy (64). Furthermore, the transcription factor Relish can regulate the expression of autophagy-related genes in *T. molitor* through unknown mechanisms. This was briefly addressed in a study wherein silencing of *Tm*Relish in *T. molitor* larvae decreased the mRNA levels of *Tm*Atg1 in the fat bodies and hemocytes subsequent to *Listeria* infection (47). This proposes a cross-talk between *Listeria*-induced autophagy and Imd pathway in *T. molitor* (63).

## Final Remarks

We have provided a comprehensive overview of the Imd signaling cascade in *T. molitor* and insights into future research directions that would improve understanding of this signaling cascade in beetles. The existing genome sequencing information have identified players associated with the Imd signaling cascade; however, several aspects remain unanswered. These include (*i*) the precise mechanism of the Imd pathway compartments such as FADD, Dredd, TAK1, and TAB2, (*ii*) cross-talks with other signaling pathways, such as Toll, JNK, and autophagy, and (*iii*) the putative functions of this signaling in development and apoptosis, similar to its counterpart, TNFR signaling, in mammals. Therefore, further studies are essential to bridge these gaps in the literature.

## Author Contributions

YH and YJ: design manuscript concepts. MA and HJ: wrote the draft manuscript. MA, HJ, and YJ: wrote the manuscript. BP, YH, and YJ: revised the manuscript. All authors contributed to the article and approved the submitted version.

## Funding

This research was supported by Basic Science Research Program through the National Research Foundation of Korea (NRF) funded by the Ministry of Science, ICT and future Planning (Grant No. 2019R1I1A3A01057848) and Korea Institute of Planning and Evaluation for Technology in Food, Agriculture and Forestry (IPET) through Agricultural Machinery/Equipment Localization Technology Development Program, funded by Ministry of Agriculture, Food and Rural Affairs (MAFRA) (no.321055-05).

## Conflict of Interest

The authors declare that the research was conducted in the absence of any commercial or financial relationships that could be construed as a potential conflict of interest.

## Publisher’s Note

All claims expressed in this article are solely those of the authors and do not necessarily represent those of their affiliated organizations, or those of the publisher, the editors and the reviewers. Any product that may be evaluated in this article, or claim that may be made by its manufacturer, is not guaranteed or endorsed by the publisher.
